# Measuring the Level of Urban–Rural Integration Development and Analyzing the Spatial Pattern Based on the New Development Concept: Evidence from Cities in the Yellow River Basin

**DOI:** 10.3390/ijerph20010015

**Published:** 2022-12-20

**Authors:** Leiru Wei, Xiaojie Zhao, Jianxin Lu

**Affiliations:** 1 School of Economics and Management, Zhengzhou University of Light Industry, Science Avenue 136, Zhengzhou 450000, China; 2 School of Finance, Zhongnan University of Economics and Law, Nanhu Avenue 182, Wuhan 430073, China

**Keywords:** urban–rural integration, new development concept, sustainable development, the coupling coordination

## Abstract

Urban–rural integration development (URID) is the solution to the excessive urban–rural gap, unequal and insufficient development in urban–rural areas, along with the process of dynamic and balanced urban–rural growth. The promotion of high-quality development and sustainable development in the Yellow River Basin (YRB) depends heavily on the scientific development of an evaluation index for urban–rural integration (URI), the quantitative measurement of the level of URI, and the accurate identification of the spatial layout of URI. The URI indicator system is built using the new development philosophy, and 94 cities in the YRB are used as samples. The spatial and temporal evolution characteristics of the URID in the YRB were studied from 2010 to 2020 using the entropy value method and coupled coordination model. The study shows that from 2010 to 2020, along the YRB, both urban and rural development (URD) levels generally increased. However, regional differences increased and development levels varied, showing a trend of uneven development between provinces. Overall, the degree of URID was increasing and still low, but there are three main types of urban–rural coupling and coordination (URCC) that are relatively stable: barely coordinated, primary coordination, and on the verge of disorder. Primary coordination replaced barely coordinated as the dominant type over time. Finally, it is suggested that urban and rural regions should not be “managed separately” but rather should be viewed as a cohesive organic whole; to drive urban cluster construction and spur rural development, to further close the urban–rural divide, reliance on the city centre is necessary. Concurrently, this encourages the transfer of farm labour and supports the coordinated growth of urban–rural industries; investment in advantageous industries is strengthened; the construction of URIs should be promoted at a more microscopic city and county level; and strong support is provided to achieve high-quality sustainable development of the YRB. It is important to put into practice the new development philosophy, investigate the fundamental causes of the growing urban–rural divide, change the development strategy, and optimize this new development path.

## 1. Introduction

China has made tremendous progress in economic growth and social development since the reforms, and land use, industrialization, employment and social structures in both urban and rural regions have notably changed [1]. The urban-biased development strategy, the citizen-biased distribution system, and the heavy industry-biased industrial structure—all of which were influenced by the theory of “economic dualism”—have given rise to the “three divisions” of the urban–rural divide, land division, with the separation of people from the land, further worsening the disparity between URD [2]. In the 21st century, China used a number of major strategies to coordinate the URD to address the “three rural problems” [3] and the unequal distribution in both urban and rural regional development, including urban and rural coordinated development (URCD), the creation of a new socialist countryside, precise poverty alleviation, new urbanization, and urban–rural unity (URU). However, the overall growth and effectiveness were not obvious, and some contradictory strategies were employed [4]. Since the 18th National Congress of the Party, China’s economy has faced serious and complex domestic and international risks and challenges. On the one hand, international trade is in the doldrums, economic globalisation is experiencing headwinds and the external environment for economic development is poor; on the other hand, socialism with Chinese characteristics has entered a new era, social conflicts have transformed into conflicts between people’s growing need for a better life and unbalanced and inadequate development, and economic development has shifted from high-speed growth to high-quality development. Economic development has shifted from high-speed growth to a high-quality development stage. In the face of the new contradictions and problems of the new era, China has profoundly grasped the world development trend, made scientific judgments on the world economic trend and China’s economic situation, innovatively put forward a series of theories and concepts, and formed the economic thought of socialism with Chinese characteristics in the new era. This important thought, with the new development concept of innovation, coordination, green develpment, openness and sharing as its main content, was put forward on the basis of a profound summary of domestic and international development experience and an intensive analysis of the general situation at home and abroad, and is a key and vital thought to help cross the new normal and gradually achieve high-quality development. China has now started a new era characterized by high-quality development. It is also dealing with a number of unbalanced and uncoordinated issues related to social and economic growth. URI helps to address the issue of socialist contradictions. The new development concept is derived from a summary of economic development experiences, a profound reflection on the contradictions and problems that exist in economic development, and an important idea to guide high-quality economic development. URIs must also be guided by this new development philosophy, with the goal of achieving a balanced allocation of resources and comprehensive, well-coordinated, and sustainable economic and social development in both urban and rural regions and establishing a URI indicator system. With a large population, a diverse ecological environment, and an uneven distribution of natural assets, including human and technical resources concentrated in the middle and lower reaches and energy and mine resources stored in the midstream and upstream reaches, the YRB is a significant eco barrier and an economically important area in China. The socioeconomic characteristics of the top, medium, and low reaches clearly differ from one another in most cities lagging behind in economic development and a significant divide between cities and the countryside. It is particularly important to close the gap between town and country in the YRB and always to achieve high-quality development for balanced, coordinated, and sustainable city and rural growth in the setting of high-quality development in a new era. To achieve this, this study applies a coupled coordination model and the entropy value approach to assess the degree of URIs in 94 cities within the YRB and to identify the characteristics of their geographical and temporal patterns. To this end, this study adopts the entropy value method and coupled coordination model to evaluate the degree of URI development in 94 cities in the YRB and reveal the evolution characteristics of their spatial and temporal patterns, with a view to implementing in-depth policies and measures for URIs in the YRB, laying the foundation for further discussions on the variables influencing the degree of URIs, and providing data support and some reference for successful URI initiatives for other provinces and cities.

## 2. Literature Review

As urbanization continues to accelerate, factors of production, such as capital and labour, are constantly gathering from the countryside to cities. The drawbacks of the urban–rural dual dichotomy are becoming increasingly obvious, affecting sustainable economic and social development. Globally, scholars have conducted in-depth research with rich results on URID from a variety of angles, mainly focused on the theoretical interpretation of URID, influencing factors, evaluation index system construction, and level measurement, etc.

### 2.1. Study of the URI’s Connotation

The urban–rural relationship, which is the essential economic and social relationship in human social evolution, is a dynamic and coexisting relationship between town and countryside that affects, interacts with, and restrains one another [5]. The focus is on the human, geographical, economic, social, and ecological characteristics of town and country regions [6,7]. With respect to the theoretical aspects, in the West, the issues of urban–rural relations and how to coordinate URD have historically been a concern, mostly with the concepts of “urban–rural linkage” and “URD”, but rarely references “URI”, and the relevant studies are more diffuse, diversified and complex, but all fall within the realm of urban–rural relations [8]. Ideal socialism is an ideology dating back to the 16th century, which introduced the concept of URD and served as a source of inspiration for later research on urban–rural connections [9]. Marx and Engels critically inherited the idea of URD from idealistic socialists and first introduced the concept of “URI”, dividing urban–rural relations into three stages: urban–rural confrontation, accelerated separation of the town and country area and URI, pointing out the fact that the inescapable tendency of URD is URI [10]. Since then, more scholars have conducted theoretical studies on urban–rural relations from different dimensions, resulting in theories such as the “urban bias theory”, “urban–rural dichotomy”, “urban–rural co-development”, and “rural bias theory” [11]. In practice, Germany has given priority to the idea of a balanced spatial reconstruction of urban and rural areas and has put forward the concept of urban–rural equivalence, considering urban and rural areas as a unity and promoting a “different but equal” quality of production and life in urban and rural areas on the basis of respect for the objective differences between them. Subsequently, the European Union also adopted the concept of equivalence as one of the policies that constitute the problems of uneven urban–rural development and regional rural governance, and China also learned from the Western concept of “urban–rural equivalence” to solve some of the problems caused by rapid urbanization [12].

China has long maintained an urban–rural dichotomy, and there is a substantial discrepancy in the level of URID. To overcome the persistent problem of urban–rural separation and land division, along with the separation of people and land brought on by the city’s biased development strategy, the citizen’s biased distribution system and heavy industry’s biased industrial structure advance the ideas of urban and rural coordination (URC), URU, and URI one after the other [13]. Since then, the spatial layout of towns and cities has been continuously optimized, the capacity for sustainable development has been enhanced, and the institutional mechanism and policy system for integrated URD have been basically established. URI and co-prosperity can be attained through URID, which is fundamentally an inheritance and sublimation of the concepts of URU and integrated urban–rural development. To some extent, URC is an important means, URU is the ultimate goal, while URI is a state and process. In general, they clearly state the principles of inseparability, positive interaction, deep integration, and shared prosperity between urban and rural communities [14,15]. This enhances understanding of urban–rural interactions as well as the development of concepts from earlier works that is the target orientation of urban–rural relations. The academic community has gradually begun to conduct research on URI on the basis of defining the relationship between these fundamental ideas. Various viewpoints have different interpretations of what the term “URI” means. Sociologists prioritize the development of urban–rural ties and hold that URI is a natural outcome of the growth of these relations [16]. Economists mostly use the logic of political economy to explain urban–rural integration with regards to production, allocation, exchange, and consumption. The “scissor difference” between urban and rural products can be reversed by stimulating the market mechanism and maximizing economic benefits for both urban and rural regions, fostering communication and cooperation in both the agricultural and non-agricultural sectors [17]. According to systems theory, the town and country system is an organic whole made up of the urban and rural subsystems and is distinguished by plurality and complexity. It is believed that URI is not only the integration of urban–rural economies, but it also includes the coupled and coordinated development of multidimensional subsystems, such as population, space, society and environment, ultimately forming a complex urban–rural system with complementary functions and shared benefits [18]. Economic geography puts a strong emphasis on spatial research, concentrating on the natural articulation and URI spatial subdivisions. In addition, town and country territorial systems are taken as the basic research object for the study of URID in geography, with URID research characterized by comprehensiveness, territoriality and intersectionality growth [14].

### 2.2. URI Evaluation Index System Construction

The promotion of URID requires an accurate grasp of the current situation of URID and the formulation of appropriate policies for different stages of development. As a result, scholars’ research is focused on developing an indicator system to measure the degree of URID, which is primarily reflected in the shift from one-dimensional to multidimensional evaluation indicators, from qualitative to quantitative evaluation methods, and from macro to micro research scales. First, the selection of research indicators has developed from one-dimensional to multidimensional. Research on “urban–rural integration” conducted by foreign academics has a narrow evaluation objective. It mainly focuses on a single perspective, such as the urban–rural economic gap [19], urban–rural human capital [20], urban–rural industrial integration [21], urban–rural agricultural development, urban–rural public services [22,23], and urban–rural welfare [24], and focuses on the deep-rooted problems of the current URCD in Western countries. In contrast, there are two broad ideas for the construction of an indicator system for quantitative measurement of the degree of URID in China: In one type, the urban–rural geographical system is studied as an organic whole, mostly adopting urban–rural comparative indicators, while the other is to select the corresponding comprehensive indicators from the urban and rural subsystems separately for evaluation, both of which have common features in terms of representational meaning. Chinese academics have conducted systematic research on URI from a multidimensional perspective on the basis of this. The majority of academics agree that the key factors in creating a URI evaluation index system are economic, social, and demographic integration [25,26]. However, as socialism with Chinese characteristics enters a new age and the population’s demand for a better life increases, elements such as infrastructure, public services [23], the ecological environment [26], and cultural concepts are constantly introduced into the evaluation system of URI. Town and country economic, social, spatial, population, ecological, infrastructure, along with living integration, are some of the elements that scholars have started to incorporate into evaluation index systems for URIs based on the concept of integration [27]. For example, the construction of the URI evaluation index system occurred in five dimensions: demographic, spatial, economic, social and ecological environment, as a basis for the analysis of the current situation of different URIs, and suggestions for further strengthening the degree of URIs [4]. Evaluation of URI at the national level and research and analysis of URI includes differences between regions in the temporal and spatial dimensions [28,29]. Second, the evaluation method changes from qualitative to quantitative. In foreign countries, the evaluation of URIs was dominated by qualitative analysis that constructs an interpretive framework. For instance, a paradigm for the URI of space was presented in response to the phenomena of unequal economic benefits per capita of town and country residents, and then the historical connection of town and country environments was investigated [30]. Starting from the perspective of the regional policy context and a summary of practice patterns, the local diversity and specificity of URIs in Latvia, Europe, was analysed [31]. The research on China’s URID mostly adopts quantitative methods for evaluation, including the comprehensive index method [27], coupling coordination degree model [32], fuzzy comprehensive analysis method [33], geographical weighted regression analysis method [25], and social network analysis [34], etc. In terms of the frequency used for quantitative evaluation, the integrated index method and the coupled coordination model were mostly used, which reflects the concepts of system theory and binary structure theory. The determination of weight is a key point in the comprehensive evaluation, which mainly adopts the entropy method [33], analytic hierarchy process, improved TOPSIS method [4], and principal component analysis method [25].

With further research into the space–time evolution of URIs and the factors influencing them, the use of spatial data has placed greater demands on evaluation methods, and spatial statistical methods are being used more frequently, such as global principal components analysis instead of the classical principal component analysis method, to ensure that the evaluation includes results on the dimensions of unity, integrity and comparability [35]. The third is the evaluation index from macro to micro focus. The existing research scale in China is dominated by macroscopic scales such as national, urban clusters, economic zones, provinces and cities [25,27,36,37]. Hot research areas are mainly focused on the capital ring region, the Pearl River Delta [35], the Yangtze River Delta [38], Huaihai Economic Zone [37], Jiangsu [39], Zhejiang [40] and other economically developed provinces. Some scholars have analysed the overall characteristics of URIs from the provincial level based on the macroscopic scale and usually use cluster analysis to classify the URI evaluation results into classes and then further analyse the evolution of spatial and temporal patterns [27]. From the perspective of time scale, the existing research has changed from static research on the sectional data of a node, to dynamic research on panel data. From the use of the sectional data of a province in a certain year, measurement and analysis of URD levels and differences between regions [4] has changed to utilizing panel data to measure and analyse the spatiotemporal evolution of URD levels in Chinese provinces [41].

### 2.3. A study of the Factors Influencing Urban–Rural Integration

Scholars’ attention is increasingly being drawn to the URID, how to make the URID more coordinated, and the elements that drive urban–rural integration development. Foreign research themes focus on both urban–rural factor mobility and urban–rural income disparity, mainly including population migration, land transfer, capital to the countryside, etc. Among them, foreign geographers have explored more about the urban–rural factor flow, and some of them have studied the role of migration, ethnic and cultural exchange and resource allocation in the development of URIs, reflecting, in part, the complicated social and political history of Western nations. China pays particular attention to the “three rural issues” and urban and rural residents’ quality of life. The existing research mainly focuses on the system perspective, factor perspective and the perspective of the main in-depth discussion. Based on a systems perspective, the impact of various subsystems, such as the natural environment, space, economy, social needs, culture and policies and institutions, on URID is explored, in which the discussion of the connection between the various systems typically combines qualitative and quantitative techniques [27]. There is a factor-based perspective that focuses on the impact of the spatial heterogeneity and unevenness of specific factors, such as population, capital, transportation, infrastructure, science and technology, and public services, on the growth of URIs. For instance, this study attempts to examine the impact of the workforce, lands, capital, industry, and transportation on the growth of URIs in the context of the elements based on the execution of rural revitalization strategies [42]. A subject-based viewpoint is another approach, and it focuses on how government, farmers, businesses, and other subjects’ behaviour affects the process of URID. While the government decides on local financial investment and the establishment of pertinent policies to benefit agriculture, migrant workers are affected by their own occupation, monthly income, age, level of education, and relatives, which produce differences in the choice of local urbanization and reshoring location, the location of enterprises, industrial interaction, farmland management, etc. These have a direct impact on the construction of town and country industrial chains and town and country economic integration development levels [8]. URID is a process by which the relationship in both urban and rural regions evolves in a coordinated manner with strong dynamics, uncertainty and a complex synthesis. The commonly used quantitative research methods are a principal component analysis, the Markoff chain, the geographic detector, a Pearson correlation test and so on [18]. The research results on the URID at the domestic and international levels are relatively fruitful; however, studies that build assessment indicators for urban–rural integration based on the new development philosophy are less common. The YRB has made progress towards ecological preservation and high standards of development, but research on the space–time distribution of URID in the region is still in its infancy. To this end, from the perspective of the new development philosophy, the evaluation index system of URID at the municipal level in the YRB is constructed from five dimensions: integration of innovative development, integration of coordinated development, integration of green development, integration of open development and integration of shared development. By using the method of entropy and measuring and analyzing its evolution, the paper reveals the spatiotemporal developmental pattern of its level of development for URI, which effectively supplements the existing research, with a view to providing reference for research on URID in academia and providing reference for the practice of national and regional URID.

## 3. Indicator System Construction

### 3.1. Study Area

**Figure 1:**
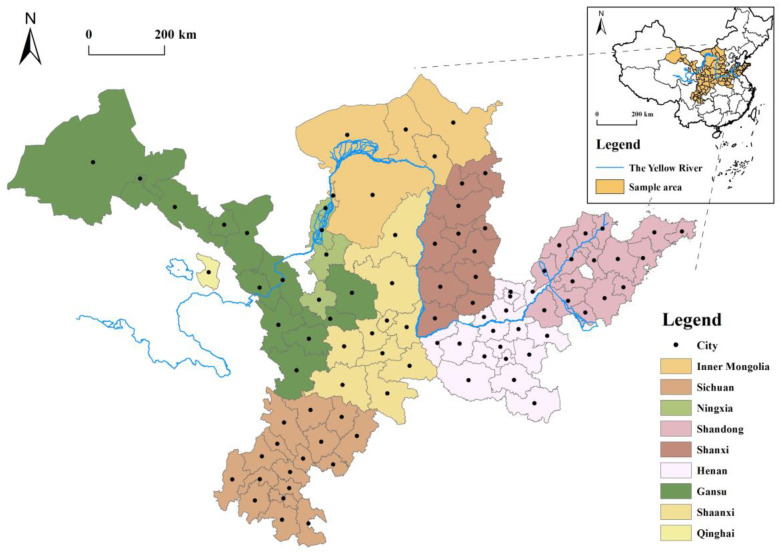
Study Area.

The YRB is the mother river of the Chinese nation. It is located at latitudes 31°31′~43°31′ N and longitudes 89°19′~119°39′ E. It comes from the Bayan Har Mountains in the Chinese region of Qinghai, straddling the Tibetan Plateau, the Inner Mongolia Plateau and North China Plain. The river passes through the provinces of Shandong, Gansu, Shaanxi, Inner Mongolia, Qinghai, and Sichuan. Its overall length is 5464 km, and its watershed area is 795,000 km^2^ (including 42,000 square kilometres of instream area). The YRB (Figure 1), a large ecological corridor that crosses three vital regions in China’s east, centre, and west, represents a significant energy and heavy industry hub. The geomorphology and natural environment of the upstream, middle and downstream regions are quite different, including various ecosystems, such as grassland, forest and wetland, and the natural system, which is complex and vulnerable. In particular, the Loess Plateau ecological environment is under pressure, and its downstream soil erosion is serious, constraining regional URD, so that most regions in the basin are relatively backwards in terms of economic development and URD disparities.

In 2020, the urban population of nine provinces in the YRB accounted for approximately 59.5 percent, less than the national urban population of 63.8 percent. The level of industrialization in the basin is generally low, and industrial modernization is insufficient. The average tertiary sector of the economy is approximately 50%, lower than the national average. It is crucial to address the issue of uneven and insufficient development of the YRB in the context of increasing urbanization, and rural rehabilitation due to “ecological protection and high-quality development of the YRB”, a significant national goal.

### 3.2. Construction of the Index System

The countryside and the city coexist and are interdependent, interwoven, and mutually beneficial. URI is a multilevel, multidomain and all-round comprehensive integration concept that includes economic, spatial, social and environmental integration [1]. This new development philosophy, as the action guide and basic directions for development in the new age, is a sharp tool to solve unbalanced and insufficient development and has emerged as an important guide for addressing the disparity in both town and country regions and the insufficient development of country areas. Areas that need to be addressed include: the innovation to address the issue of inadequate capabilities; coordination to solve the imbalance between city and countryside development; green development focusing on the issue of sustainable development; openness focusing on the issue of space for development; shared development, as the essence of socialism and the issues of social equity and justice. The aim is to build a comprehensive evaluation index system of URI growth from the five dimensions of innovation, coordination, green development, openness, and sharing that comprises two subsystems for the urban and rural development levels. Among them, the subsystem of the urban development level contains 13 basic indicators, and the subsystem of the rural development level selects 10 basic indicators. As an evaluation value for the degree of coordinated URD, the index system uses the degree of coupling and coordination in both urban and rural systems (Table 1).

Innovation gives impetus for development. Technological innovation has a leading and supporting role in solving the problem of urban–rural integration, where financial investment provides strong support for technological innovation, and increased investment helps the development of technological innovation, and the increase in the number of patents granted as the output of technological innovation reflects the progress of technological innovation. The innovation subsystem of the urban development system includes technology capital investment and scientific and technical outputs [41]. China’s total level of science and technology is lower than that of developed nations, and the country’s development momentum is insufficient for innovation. However, the improvement of science and technology investment and the increase in the number of patent authorizations reflect the great support of science and technology in China, which breaks the monopoly of Western countries and addresses the problem. Technological innovation in the countryside is mainly reflected in the increase in the level of modernization of agriculture, which is the replacement of labour by mechanical power and the increase in food production while improving labour efficiency. In the innovation subsystem of the countryside development system, two indicators were selected: the total power of agricultural machinery per unit of arable land and the growth in food production [4].

Addressing development disparities is the main goal of coordinated development, mainly including population coordination, industrial coordination and living standards coordination [43]. Since the reform and opening up, China’s economic policy has long inclined towards the cities, which have an industrial structure dominated by secondary and tertiary industries, providing a large number of employment opportunities for residents, while the countryside is dominated by primary industries and the added value of agriculture is low, and the difference between urban and rural industrial structures has delayed the economic development of the countryside. The accelerated urbanization process has led to the “hollowing out” of the countryside, and the burden of urban population is becoming more serious, with the demographic structure becoming out of balance. Finally, improving the living standards of urban and rural residents and reducing the gap between them is one of the goals of urban–rural integration. Therefore, the urbanization rate, urban dwellers’ per capita disposable income and the Engel coefficient of urban inhabitants were selected in the coordinated urban development subsystem. The former reflects the population coordination within city and countryside areas. The latter two represent the standard of living and the quality of life of city dwellers, respectively. The coordinated rural development subsystem selects the synchronous development degree of industries, country dwellers’ per capita disposable income and the rural dwellers’ Engel coefficient of rural residents [44]. The former reflects the optimization of the economy’s industrial structure, and when its value is zero, the duality of economic structure is the most significant. When it is one, the duality of the economic structure disappears. The latter two can be used to gauge rural dwellers’ quality of life and standard of living.

Green development focuses on concerns of sustainable development and seeks to establish a harmonious balance between people and the natural world. The ecological environment in urban and rural areas is an important support for the URID. On the basis of ecological protection, it is necessary to promote the improvement of the ecological environment and the efficient use of resources in urban and rural areas, so as to achieve equal and sustainable development in urban and rural areas. On the one hand, the level of greening and environmental management in urban and rural areas affects residents’ lifestyles and feelings about living. The urban green development subsystem selects the green coverage rate, industrial sulphur dioxide emission rates and the percentage of residential waste in the built-up region that is safely treated [45]. The percentage of green coverage in the built-up region reflects the ecological environment protection degree of the town. China’s population is enormous, but its resources are scarce, and economic development has made environmental problems increasingly worse. The reduction in industrial sulphur dioxide emissions reflects the efficient use of resources to protect the environment; the harmless disposal rates of household garbage reflects the ability of cities and towns to protect the environment [43]. On the other hand, pollution control plays an important role in enhancing the carrying capacity of the ecological environment and adhering to green production, which is measured by the fertilizer use intensity in the effective irrigated area selected for the rural green development subsystem [27].

Open development focuses on the flow of factor resources between urban and rural areas, and the sharing of factor resources between urban and rural areas and the elimination of urban–rural barriers are important ways to achieve urban–rural integration. The issue of internal and external linkage, urban and rural transport and information infrastructure are both the basic conditions for the integrated development of urban and rural areas and the carrier for realizing the flow of factors between urban and rural areas. Building a rational urban–rural cyberspace system involves accelerating and strengthening urban–rural spatial mobility and improving information networks. The city openness subsystem includes the density of the transportation network and the density of the internet [29]; the former is represented by the ratio of highway mileage to administrative area, and the latter is represented by the ratio of internet-connected homes to all the homes in the district. The rural opening subsystem includes the number of buses per million people which represents the accessibility of rural transportation. The more buses per million people there are, the more convenient the transportation will be [46].

The goals of shared development include social justice and equity, improving people’s well-being, enhancing happiness, paying attention to enjoying homogeneous development opportunities and development rights in social services and welfare security, equalizing public service access, and reducing the gap in both town and countryside locations [25]. Thus, the urban sharing subsystem includes the number of insured personnel of urban basic medical insurance, the ratio of full-time teachers to one million students in urban compulsory education and the electricity consumption of urban residents per million people. The level of urban public services is measured from the allocation of medical care, education and infrastructure. The shared development subsystem of the rural development system includes the quantity for rural health care facilities, the quantity for people with minimum living allowances and the electricity consumption of rural residents per million people. The number of rural medical institutions can reflect the situation of rural medical facilities [47]. The decrease in the number of recipients of the minimum subsistence allowance shows that the basic life of rural residents is improving. The electricity consumption per million rural residents reflects the availability of infrastructure.

## 4. Data Sources and Research Methods

### 4.1. Data Sources

With the natural YRB as the basis, considering the integrity of the study unit and the principle of direct correlation between regional development and the YRB and drawing on relevant research results, the nine administrative provinces where the YRB flows—Qinghai, Sichuan, Gansu, Ningxia, Inner Mongolia, Shaanxi, Shanxi, Henan, and Shandong—are referred to as the study area, but in view of the lack of statistical data on some evaluation indicators in some areas, the final 94 cities were involved. Combining tradition and taking into account the completeness of the provinces, the YRB is separated into the upstream, middle, and downstream sections in Qing-Long-Ning-Meng, Shan-Jin, and Yu-Lu. The new development concept was first proposed in 2015. Combined with the five-year planning ideas, data from 2010 and 2020 were additionally selected to examine how URIs changed both before and after the new development philosophy was introduced. The original data on per capita disposable income in city and countryside areas, urbanization rate, traffic network density, urban internet penetration rate, grain growth rate, and total agricultural machinery power in relation to area in the indicator system are obtained from provincial statistical yearbooks of the corresponding years. The China Urban Statistical Yearbook along with the Economy Prediction System (EPS) database for the relevant years provided the original data for the remaining indicators, and usually, linear or mean interpolation is used to fill in missing data. It should be noted that despite the different sources of data, there is compatibility between these data and the macro data from the National Statistical Office (NBS).

### 4.2. Research Methods

The coordinated development evaluation index system for the URI in the 33 indicators data, urban development index, rural development index, and the degree of URI are each separately calculated using the comprehensive index calculated method after determining the weights of each indicator using the entropy weighting method. Finally, the coupling degree of the coordinated URD is calculated by using the coupling degree evaluation method, and the coordination degree is used to evaluate the coordination degree of the URI in the YRB.

#### 4.2.1. The Entropy Method

An assignment method based on the information entropy concept is the entropy value method, which has the advantage of objectivity. The method represents the uncertainty and variability of indicators according to information entropy’s measurement of information volume. The degree change of the indication is inversely correlated with the information entropy value, the influence of the indicator on the overall evaluation, and the weight given to the indicator. Many scholars use it in comprehensive evaluation. Due to the different units of measurement among the evaluation indicators, the data were standardized, and weights were determined using the extreme value method to better reflect the implications of positive and negative indicators. Finally, the urban development index and rural development index were measured on the basis of the linear fitting formula, and these are the precise calculation steps.

First, the original index matrix was constructed. With m  cities and n evaluation indicators, the original indicator matrix is $X={\left\{x_{ij}\right\}}_{m\times n}\left(1\leq i\leq m,1\leq j\leq n\right)$. In this study, m  represents the 94 study cities in the YRB, and n  represents the 23 urban–rural integration evaluation indicators.

The second step is indicator standardization. Positive indicators (the better the indicator, the higher its worth) and negative indicators are included in the basic indicators of each subsystem of the assessment index system of URI and coordinated development (the lower the indicator, the higher its worth). The extreme value method is used to dimensionless size the data of each indicator to create the matrix $X_{ij}$, avoiding the impact of the scale difference on data processing, with the following formula:

(1) $$X_{ij}=\frac{x_{ij}-min\left\{x_{1j},x_{2j},\ldots ,x_{nj},\right\}}{max\left\{x_{1j},x_{2j},\ldots ,x_{nj}\right\}-min\left\{x_{1j},x_{2j},\ldots ,x_{nj},\right\}}$$

(2) $$X_{ij}=\frac{max\left\{x_{1j},x_{2j},\ldots ,x_{nj}\right\}-x_{ij}}{max\left\{x_{1j},x_{2j},\ldots ,x_{nj}\right\}-min\left\{x_{1j},x_{2j},\ldots ,x_{nj},\right\}}$$

The third step is levelling. Because no 0 or 1 can appear in the indicator data when calculating weights by the entropy method, the indicator normalization interval was contracted to [0.002, 0.996] by the following formula:

(3) $$x_{ij}^{\prime }=\left(0.996-0.002\right)X_{ij}+0.002$$

Fourth, the indicators were normalized.

(4) $$P_{ij}=\frac{x_{ij}^{\prime }}{\sum_{i=1}^{m}x_{ij}^{\prime }}$$

Fifth, the *j*th indicator’s value was calculated: in which *k* = 1/ln(m*n)

(5) $$E_{j}=-k\overset{m}{\underset{i=1}{\sum }}P_{ij}lnP_{ij}$$

Sixth, the *j*th indicator’s entropy weight was calculated:

(6) $$W_{ij}=\left(1-E_{j}\right)/\overset{n}{\underset{j=1}{\sum }}\left(1-E_{j}\right)$$

Finally, the urban development index $U\left(x\right)$ and the rural development index $R\left(y\right)$ were measured according to a linear fitting formula, with the following formula:

(7) $$U\left(x\right)=\overset{m}{\underset{i=1}{\sum }}a_{i}x_{i}\left(i=1,2,\cdots ,13\right)$$

(8) $$R\left(y\right)=\overset{m}{\underset{i=1}{\sum }}b_{i}y_{i}\left(i=1,2,\cdots ,10\right)$$

where i=1,2,⋯,13 in Equation (7), which represents the 13 indicators indicating the level of city development. In Equation (8), i=1,2,⋯,10 represents the 10 indicators indicating the level of country development; $a_{i}$ and $b_{i}$ are the standardized values of the corresponding indicators of the city development level and countryside development level, respectively, and $x_{i}$ and $y_{i}$ are the normalized values of the corresponding indicators for the URD levels, respectively.

#### 4.2.2. Coupling Degree Model

A process known as coupling is when two or more physics systems interact and affect one another to the point of unification. The detailed calculation steps are as follows:

(1) Degree of development calculation $\left(\mathrm{UR}\right)$

The development index UR is a composite index of URD and can be used to reflect the combined benefits of URD. The function is:

(9) $$\mathrm{UR}=aU\left(x\right)+bR\left(y\right)$$

In the formula, a and b are coefficients to be determined, but it is generally considered that town development and countryside development have equal contribution values, so a = *b* = 0.5 in Formula (9); $U\left(x\right)$ and $R\left(y\right)$ indicate the urban development index and rural development index, respectively.

(2) Coupling level $\left(C\right)$

Urban and rural development are dependent on one another and can be measured by coupling, which is the degree of coordination of movement of two or more systems. The detailed calculation steps are as follows:

(10) $$C=2\sqrt{\left[U\left(x\right)\cdot R\left(y\right)\right]/{\left[U\left(x\right)+R\left(y\right)\right]}^{2}}$$

where $C\in \left[0,1\right]$. When C=1, it reflects that urban–rural development has reached the optimal coupling state; the closer C is to 0, the lower the correlation between cities and country areas.

(3) Degree of coordination calculation $\left(D\right)$

The most important characteristic of the degree of coordination is its high stability, combining the degree of development UR and the degree of coupling C, and its main role is to measure the coordinated development of URD as a whole. The function is:

(11) $$D=\sqrt{\mathrm{UR}\cdot C}$$

In Equation (9), UR is the composite development index, and C is the coupling degree. The existing research results are divided into 10 levels (Table 2).

## 5. Analysis of the Spatial and Temporal Characteristics of URD Levels in the YRB

### 5.1. Spatial–Temporal Analysis of the Urban Development Level in the YRB

**Figure 2:**
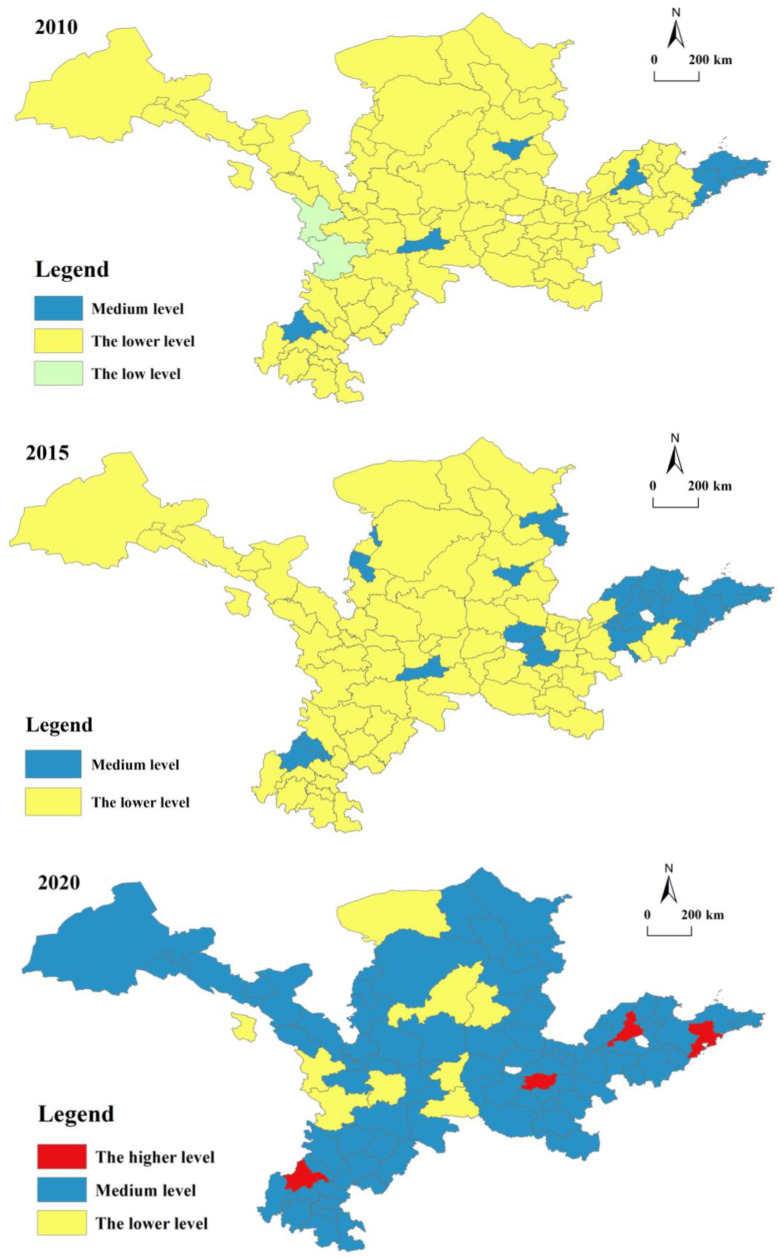
Distribution of urban development levels.

According to this calculation, the results of the urban development index and rural development index refer to existing studies [48,49] on the standards for classifying the degree of URD. This study takes 0.2, 0.4, 0.6 and 0.8 as the critical values to make spatial visualization maps for URD indices in 2010, 2015 and 2020 (Figure 2). Among them, Y ≤ 0.2 is a low development level, 0.2 < Y ≤ 0.4 is a lower development level, 0.4 < Y ≤ 0.6 is a medium development level, 0.6 < Y ≤ 0.8 is a higher development level, and Y > 0.8 is a high development level.

In Figure 2, from 2010 to 2020, the overall level of urban development in the YRB shows fluctuating growth; however, the average value of urban development in the whole YRB basically remains at approximately 0.387, while the maximum is 0.7776, and the minimum is 0.1747. This suggests that the overall level of urban development in the YRB is low and highly variable within. The types of urban development levels are relatively stable, mainly including low levels and relatively low levels. With the advance of time, the development level is transformed from a predominantly low level to a predominantly lower level, and some areas enter the medium level stage during the final part of the study period.

Combined with the characteristics of urban development changes, there are two distinct phases in the history of town development in the YRB: (1) From 2010 to 2015, the urban development level was relatively slow. The number of cities below the lower level of development decreased from 87 to 72, and the number of cities with a medium level of development increased from 7 to 22. At this stage, the new rural construction and URI were put forward, urban and rural development is further tilted towards countryside growth, and the emphasis on country economic growth is the main reason for the slow development degree of the city.

(2) From 2015 to 2020, the urban development level was relatively quick. The number of cities below the lower level of development decreased from 72 to 23, and the number of cities with a medium level of development increased from 7 to 22. Among the cities with higher degrees of development, Chengdu, Jinan, Zhengzhou, and Qingdao had perfect infrastructure, a large quantity of talent in colleges and universities, and greater levels of public services. At the same time, there was a spatial agglomeration radiation effect, which promotes the growth of neighbouring cities and a reasonably high degree of urban development.

### 5.2. Spatial–Temporal Analysis of Rural Development Level in the YRB

**Figure 3:**
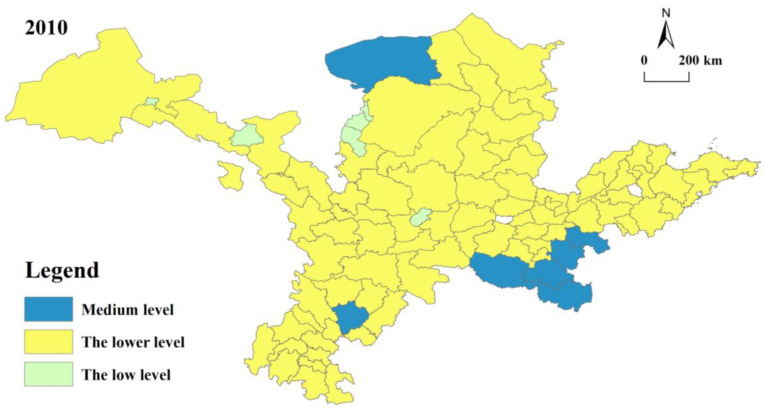

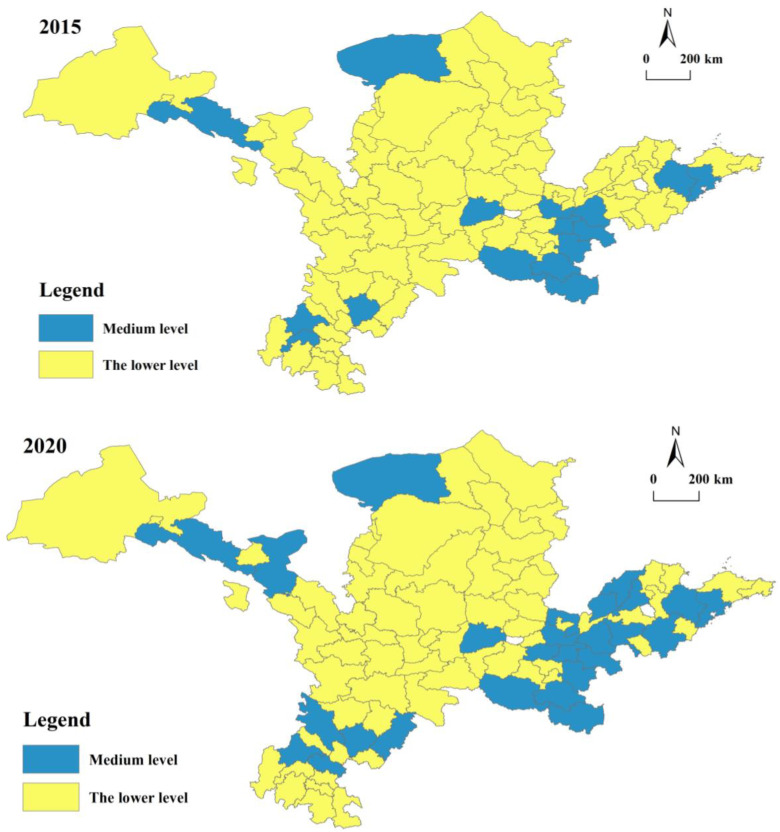
Distribution of rural development levels.

In Figure 3, in the period of 2010–2020, the degree of rural development in the YRB shows steady growth; however, the average value of rural development in the entire YRB basically remained at approximately 0.3375, while the maximum was 0.5482, and the minimum was 0.1521. This shows that the overall level of country development in the YRB is low with less internal variability. The types of rural development classes are relatively stable, mainly including medium levels and relatively low levels. With the advance of time, the development level has been mainly at a relatively low level, and no city has entered a high level until the end of this study.

Overall, rural development varies by municipality in the study area and experienced a dynamic change process of “decrement-increase”, and the regional differences increased slightly. From 2010 to 2015, the polar and standard deviations for the overall countryside growth level of the research area show a decreasing state, with the polar decreasing from 0.331 to 0.267 and the standard deviation decreasing from 0.694 to 0.632. At this stage, along with a series of strategic arrangements, such as new rural construction, URU, rural revitalization and URI promotion, the village development level was effectively promoted, and rural development levels decreased regionally. From 2015 to 2020, the extreme difference in the overall rural development level of the YRB increased from 0.267 to 0.341, and the standard deviation increased from 0.633 to 0.653, showing that the rural development level in each region tended to be dispersed and that the difference in rural development level tended to be strengthened. This stage has a greater relationship between the level of URC and the starting point of development, the regional environment, and the stage of economic development. With the deepening of URIs and the fast development in new urbanization, the differences in country growth levels among different regions are further revealed. The regions with high levels of city development have faster levels of rural development, and vice versa. The phenomenon of increasingly escalating regional inequalities in country development level in the latter half of the study period appears in accordance with the actual rural development of the YRB. By region, the trend of change in the degree of rural development in the upstream, middle and downstream of the YRB remains consistent, with smaller increases in changes in the upstream and middle, along with larger increases in the downstream.

## 6. Spatial and Temporal Analysis of the URID in the YRB

**Figure 4:**
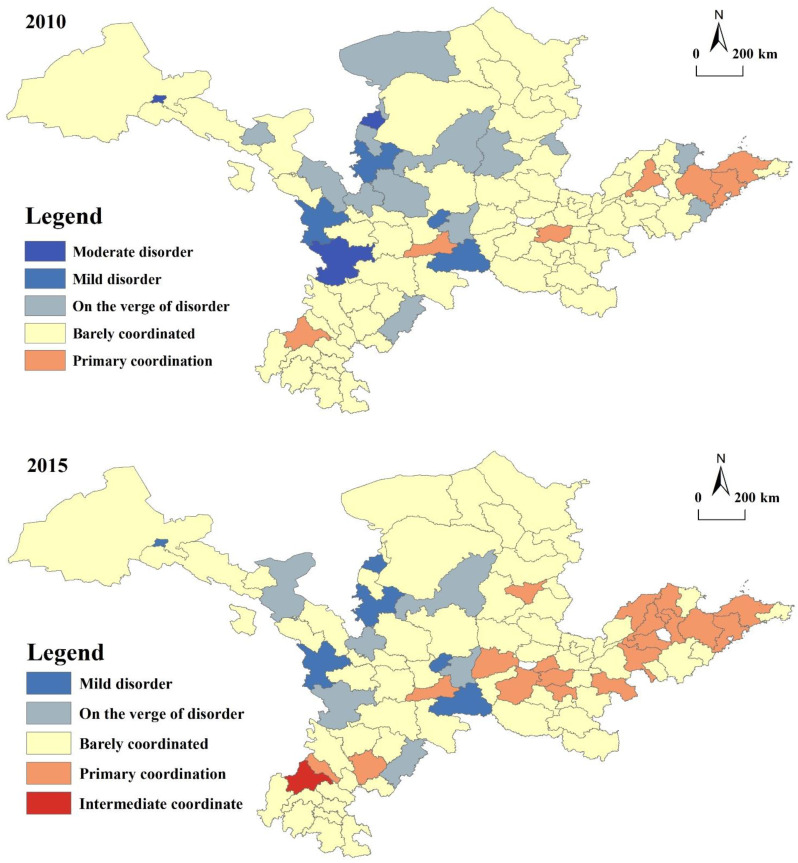

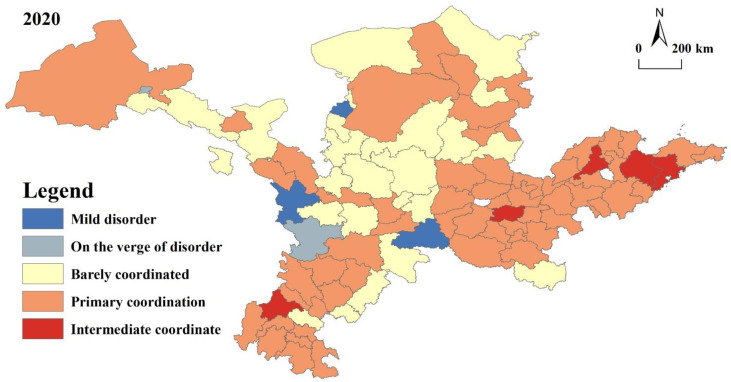
Distribution of types of URD coupling and coordination.

Based on the results calculated by the rural–urban coupling coordination index and referring to the division criteria of the coupling coordination degree from existing studies, the coordination level of coupling is divided into five major coordination levels and 10 coordination subspecies types. From Table 2, it is clear that the moderate coordination level includes two types of coordination: intermediate coordination (B1) and primary coordination (B2); basic coordination includes two types: barely coordinated (C1) and on the verge of disorder (C2); and transitional coordination includes two types: mild disorder (D1) and moderate disorder (D2). Figure 4 depicts the urban–rural linkage coordination distribution in the YRB, and the analysis is as follows.

### 6.1. Analysis of Temporal Changes in URIs

(1) From 2010 to 2020, the YRB’s level of development for each subsystem of the URI showed an upwards trend. The main manifestation is that before 2010, the country took economic construction as the central focus and rapidly promoted urbanization development. This period of URD is dominated by the one-way flow of factors from city to countryside regions. In 2010, the degree of urban–rural coupling coordination (URC) was low. From 2010 to 2015, with the deepening of the national reform of city and country relationships at this stage, the focus of the reform from the development of the “town of single direction” policy shifted to “both city and country development interests”, Changes of the policy background occurred to promote city and countryside economic and social development and ecological environment protection. Similarly, the subsystems coupling level in both city and countryside regions rose steadily, and the degree of URID became more coordinated. It is clear from this that in this chronological evolution of URI in China, with the construction of a new socialist countryside, new townization, beautiful countryside construction and a number of policies, such as precise poverty alleviation and rural revitalization, the growth of URI has a positive overall trend and shows the process of transformation from “quantitative” to “qualitative” change.

(2) The general degree of URI of development in the YRB is relatively low, but the types of urban–rural coupling coordination are relatively stable, mainly including three types: C2, C1 and B2. As time passes, the dominant type of coupling coordination degree changes from C1 to B2. From 2010 to 2015, Chengdu first entered the intermediate stage of coordinated (B1) development from B2 in 2015 and has always been the demonstration region for coordinated URD in the province. The degree of URC development in the six municipalities, including Xi’an, Zhengzhou, Jinan, Weifang, Qingdao and Yantai, has been stable at the primary stage of coordination. Except for Jiayuguan, Dingxi, Shizuishan, Wuzhong, Tongchuan and Shangluo, most prefectures in the YRB have entered the stage of basic coordinated development. From 2015 to 2020, following Chengdu, the four cities Zhengzhou, Jinan, Weifang and Qingdao, also moved from primary coordination to intermediate coordination. These cities have always been in the core zone of Henan and Shandong Provinces. The percentage of cities in primary coordination has gone from 20% to 65%. With the exception of Shizuishan, Dingxi and Shangluo, all cities along the YRB have entered a stage of basic coordinated development. This indicates that with the change in the social economy, the interaction degree of each subsystem in the YRB tends to be enhanced as a whole, but the interaction relationship needs to be further strengthened.

(3) There are interregional disparities in the degree of URI in the YRB. The characteristics of “convergence” and “differentiation” are obvious. In 2010–2015, the general degree of URI in the YRB is at the “centre, in the middle, high inside the south and low within the north, low there in the east and high within the west” with the maximum value basically appearing in the east (Zhengzhou, Jinan, Qingdao, Weifang) and the southern Chengdu, and the minimum value mainly in the upstream (Shizuishan, Wuzhong, Dingxi, Longnan). Cities in the moderately coordinated level, which have relatively high levels of coupling and development, can take advantage of the city and continue to maintain healthy and stable development at a highly coordinated level, while at the same time, driving the development of the basic coordination of the city. For cities at the transitional coordination level of which Shizuishan City has the lowest level of development, and urban–rural coupling and coordination is again the combination that reflects the level of urban–rural development both indices are low; both need further coordinated development, strengthened urban–rural two-way flow elements, mutual linkage between city and public services, and the promotion of agricultural transfer of population urbanization. In addition, the equitable exchange of production inputs and the equitable distribution of public resources between city and country communities should be addressed.

### 6.2. Spatial Differentiation Pattern of URIs

From the changes in coupling coordination in each region and the evolutionary characteristics of the value, the spatial evolution of URC development in the YRB is mapped at various time points (Figure 4).

(1) High-level coordination areas show a scattered distribution.

Figure 4 shows that high-level coordination areas are scattered in the capital cities of each province with barely any coordination as the main type of URI in the YRB. From 2010 to 2020, a total of seven cities, mainly in the three provinces of Shandong, Henan and Sichuan, have consistently maintained a primary level of urban–rural coupling coordination, namely, Chengdu, Xi’an, Zhengzhou, Jinan, Weifang, Yantai and Qingdao, with the level of coupling coordination remaining relatively high over time and showing a scattered spatial distribution. Regarding the change in coupled coordination values, the high value coordination zone within the basic coordination state evolves over time, showing a scattered distribution centred on the provincial capital cities and then gradually spreading around, with cities in Henan Province and Shandong Province accounting for up to 80% of the cities in medium coordination at the three time points. Zhengzhou city and Qingdao are the core areas of the provinces and their long-term economic development is in a leading position, at the terminal stage of urbanization, with a relatively small rural share in agriculture. Town to rural development is a spillover effect at this leading stage. Additionally, in recent years, the concept of ecological civilization and green development has been deeply ingrained within people, and these cities are at the forefront of ecological and environmental management. URIs have been effectively promoted in economic, social and ecological terms as a whole. The coordination among various subsystems has continuously improved, and the state of coordinated development among regions is sound. In the moderate coordination level of cities, these cities have a relatively high degree of coupling and development. They recognize their own city’s advantages and continue to maintain healthy and stable development with high levels of coordination. They also play a good pulling role and promote the growth of overly coordinated cities.

(2) The low-level coordination area presents a certain degree of geographical lock-in.

The long-term layout of the low-level coordination area in Ningxia and Gansu Provinces shows a certain degree of low value lock-in. In terms of type change, Ningxia as a whole was basically in a mildly disordered state in 2010, and Shizuishan city in the study area was moderately disordered. From the change in coupling coordination values, the light grey area is on the verge of a low value of dissonance, a state that has existed in Ningxia for a long time, even at the end of this study period. There were some municipalities in the region from C2 to C1, but the coupling coordination values within the scope of the study area as a whole are still at a relatively low level. The low level of coordination within Ningxia has not significantly improved. Studies have shown, that compared with the upstream regions of Gansu and Inner Mongolia, the Ningxia region’s environment is relatively poor, and the lack of natural resources and traffic inconvenience lead to industrial enterprises not being able to form scale. Since a new type of urbanization has been put forward, the Ningxia economic society has entered a period of rapid development, steadily pushing forward the urbanization process. Urban–rural relations are at a point where they are becoming polarized, and the economic and social divide in both the city and countryside is progressively growing. At the same time, due to the complex terrain in Ningxia, the agricultural industry cannot be developed, making its own industrial upgrading slow, mainly restricted to the traditional heavy and construction industries, thus inducing a series of ecological and environmental problems. Imbalance can lead to the development of URIs, and the subsystems have long had a low level of development and inadequate coordination.

### 6.3. Spatial Autocorrelation Analysis

To delve deeper into the YRB’s spatial characteristics of the degree of URI development, the global Moran-I values of 0.2245, 0.2658 and 0.2091 in 2010, 2015 and 2020, respectively, were calculated using ARCGIS software Version 10.2 and passed the test at the 99% confidence level, showing that the phenomenon of spatial clustering of URI development levels in the YRB is significant. In other words, URI is higher in nearby regions that have higher degrees of urban–rural linkage coordination, and vice versa.

**Figure 5:**
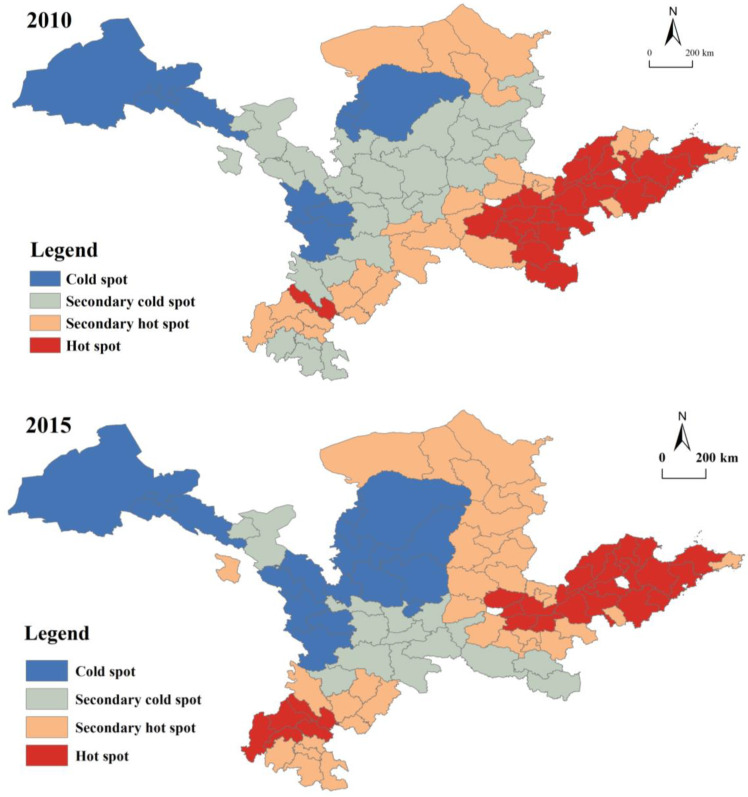

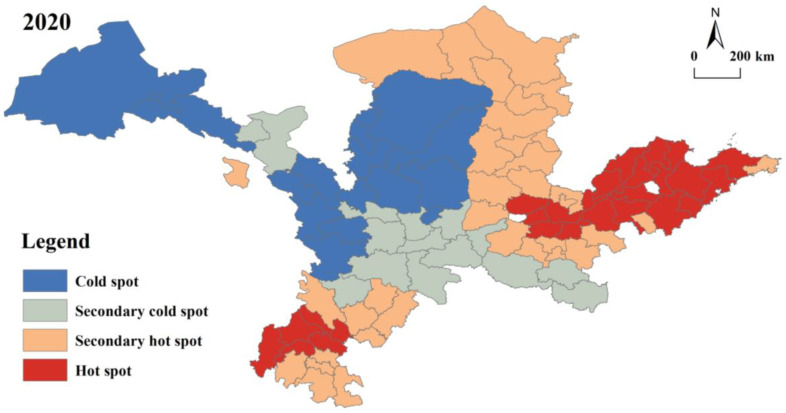
Evolution of cold hotspots of urban–rural integration.

To ascertain the spatial correlation of overall urban–rural integration, the global Moran index was used. ARCGIS software was applied to conduct a cold hotspot analysis of spatial associations to further investigate the variability of spatial autocorrelation in various cities. The significant zones of spatial correlation types were divided into four levels, hot spot, secondary hot spot, secondary cold spot and cold spot, which generated the hot spot evolution diagram of the spatial pattern of coupling coordination degree, as shown in Figure 5. The results show that 19.4%, 17.9% and 13.4% of the evaluation units show a significant positive spatial correlation in 2010, 2015 and 2020, respectively. In particular, the hotspot agglomerations are mainly located in the downstream Shandong Peninsula blue and yellow economic zone and the Central Plains city cluster, such as Qingdao, Yantai, Zibo and Zhengzhou, as well as some scattered cities in the west, such as Chengdu, Mianyang and Deyang, which have the advantages of good regional coordination and linkages, economic growth and infrastructure development through the trickle-down effect of radiation from major cities such as Qingdao and Chengdu, coupled with the gradual improvement of the regional collaborative governance pattern and a high degree of URC in the region. Sub-hotspot agglomerations were mainly distributed near hotspots and in the northern part of the YRB. In 2010, they were in the northern and southwestern border cities and spread inland. In 2015, they were mainly divided among the early midstream northeast and western regions. In 2020, they were concentrated in the Central Plains city cluster with the Chengdu-Chongqing economic circle. In 2019, the national Urban–Rural integration and development pilot area Jinan and Qingdao were in the polarization stage of absorbing various resources gathered around them, resulting in a relative lag in urban–rural integration in the less developed cities surrounding them. The cold spots are mainly located in the western part of the YRB and some midstream areas, such as Jiuquan, Jiayuguan, Zhangye, Tianshui, Dingxi and Longnan, where the construction infrastructure is weak and the regional development endowment is insufficient, constrained by the location, traffic and resources, and forming a “depression” for the development of URIs. The secondary cold spots are mainly located in the Guanzhong city cluster, spreading inland over time. The cold spots are in the western part of the YRB and in some midstream areas.

## 7. Conclusions and Implications

### 7.1. Conclusions

Using the new development concept as a guide, five dimensions make up the evaluation system for urban–rural integration, including innovation, coordination, going green development, openness, and sharing. From 2010 to 2020, the YRB’s level of urban–rural integration was measured using the coupled coordination model, and then its temporal and spatial evolution characteristics were analysed. The findings are as follows:

(1) Both urban and rural development levels are on the rise, and there is a polarization of development levels. The level of development in the midstream and downstream is higher than the development in the upstream, along with an imbalance between the two, with urban development levels being higher than rural development levels. In terms of spatial distribution, there are still some differences in the level of coupling coordination among the provinces, and the phenomenon of peripheral radiation from provincial capital cities is more obvious, mainly manifested in the development of towns and villages in surrounding cities driven by radiation from cities, such as Zhengzhou, Jinan and Chengdu.

(2) From 2010 to 2020, the degree of URI development along the YRB is on the rise and low overall, but the type of urban–rural coupling coordination is relatively stable, mainly including three types of C2, C1, and B2. Over time, B2 replaces C1 coupling as the predominant coupling type. The YRB’s regional variations in the coupling coordination of URI systems exhibit a fluctuating trend of growth, with the variation in coupling coordination between downstream and upstream being the primary reason for the variations across the basin, while the difference within the upstream is larger than the difference within the downstream. Most of the 94 cities in the whole YRB, except Shizuishan, Dingxi and Shangluo, fall under the basic coordination type, which shows that both urban and rural growth interact and impact one another and that there is a clear coupling relationship. Among the coordination levels, the intermediate and primary levels are scattered, while the others show a clustered distribution of each scatter, with low overall coordination, high internal variability and more obvious regional differences.

(3) Using the ESDA method and ArcGIS software to calculate the global Moran-I values, the degree of URI development in the YRB was found to have significant spatial clustering. Regions that have higher levels of urban–rural integration also have higher proximity, and vice versa. Specifically, the Central Plains city cluster (CPCC) (a cluster of 23 cities, including Kaifeng and Luoyang, centred on Zhengzhou, committed to becoming the largest city cluster in the central and western regions) and the Shandong Peninsula blue economic zone (SPBE) (China’s first regional development strategy with the theme of marine economy, including all the sea areas of Shandong and six cities including Qingdao, Yantai and Weifang) are the primary hotspots. Sub-hotspots are located in the Chengdu-Chongqing economic circle (CCEC) (An economic development region centred on the two major cities of Chengdu and Chongqing), along with the CPCC, which has a strong siphoning effect on neighbouring cities. Sub-cold spots are spread inland by the Guanzhong city cluster (GCC) (with Xi’an as the centre, including five cities such as Xianyang and Baoji, it is the core area of Shaanxi’s economy). The cold spots are in the western part of the YRB and some midstream areas.

### 7.2. Study Implications

First, urban and rural areas are linked in organic unity, and the cooperation of all subjects and levels has led to the region’s coordinated growth. At a more microscopic city and county level, the coordinated and integrated development of the city and countryside in the YRB should be encouraged. Some counties with better basic conditions should be selected as provincial pilot zones for urban–rural integration development along the old Yellow River Road to drive the overall improvement of the demonstration belt construction with key breakthroughs in the pilot zones and close the divide between the YRB and other regions and the urban–rural gap within it.

Second, relying on the city centre to encourage the construction of urban clusters drives rural development and further closes the town and countryside divide. The upstream, middle and downstream reaches of the YRB have uneven distributions of natural resources and large differences in the ecological environment. The middle and upper reaches are mainly in Henan and Shandong, and priority should be given to cultivating the construction of central cities, such as Zhengzhou, Xi’an, Qingdao and Jinan. With the rise of the Central Plains region, the CPCC and the Shandong Peninsula city cluster (one of the national city clusters covering all cities in Shandong Province) should play a role in driving the development of the surrounding city and country regions and reducing the distance between them. The ecological environment is fragile in Gansu, Qinghai and Inner Mongolia, and ecological protection needs to be the main focus, combining ecological protection with economic development and bringing into play the advantages of dry crop agriculture, tourism and animal husbandry. Secondly, the city cluster is a collection of cities with a high concentration of economic activity and population and a relatively complete industrial chain. Priority can be given to the development of Xining, Lanzhou, Hohhot and other key cities and then drive the support and protection role of the Lanzhou-West city cluster (the economic zone of 22 prefectures and cities, centred on Lanzhou City in Gansu Province and Xining City in Qinghai Province, and an important cross-provincial city cluster in western China), Ningxia along the YRB city group (ten cities in Ningxia, including Yinchuan, Shizuishan and Wuzhong, spread along the YRB) and the Houbao-E city cluster (including Hohhot, Baotou and Erdos in Inner Mongolia Autonomous Region and Yulin in Shaanxi Province) to the upper reaches of the river, radiating the urban and rural development in the middle and upper reaches of the YRB.

Third, investment in advantageous industries should be increased. Agricultural labour should be attracted to the region, and the integrated growth of industries in both the cities and country should be boosted. Upstream of the YRB is dominated by manufacturing and electrical industries. The nonfarm shift of the agricultural labour force is promoted through the creation of a western advanced manufacturing base. At the same time, as an important ecological reserve, it provides the full advantages of modern ecological agriculture and animal husbandry, such as dry crop agriculture, herbal medicine cultivation, grass livestock, etc., to create a plateau of a green organic food production base and moderate development of special niche tourism. Middle and downstream of the YRB are important grain-producing areas with the advantage of well-developed light industries, such as textiles and foodstuffs, which drive the modern agriculture development level by strengthening the cultivation of new agricultural business entities. Taking innovative resources as a breakthrough and increasing innovation investment, the role of Xi’an, Zhengzhou and other central cities as innovation demonstration zones is being fully exploited, encouraging cities to strengthen industrial linkages and collaborate based on their own industrial and resource advantages, bringing about the overall economic development of the basin’s cities and country regions and attracting the nonfarm transfer of agricultural labour. The aim is the creation of a modern, high-quality industrial agriculture system through the encouragement of the coordinated growth of the countryside primary, secondary, and tertiary industries.

Fourth, URI is a gradual process that is often accompanied by uneven growth of urban development and countryside revitalization with internal differences. Thus, there is a demand for research into the deeper drivers of the widening urban and rural gap as well as the implementation of URI strategies in a planned, phased and targeted manner. The new development philosophy transforms the development mode, optimizes development ideas, realizes the unification of ecological, economic and social benefits. It provides strong support for the high-quality and sustainable development of the YRB. The industrial structure and demographic transformation of the countryside should be emphasised, along with the development of agricultural industries with more regional characteristics and optimal industrial structure. The degree of social security should be increased and reform of the family registration systems enhanced to advance the civilization of the agricultural transfer population. The issue of protecting the rights and interests of the new generation of migrant workers should be addressed and coordinated development of URI realized. The absorption capacity of cities and towns for the agricultural population should thus be enhanced. Meanwhile, the training of professional work skills for the rural migrant population has been strengthened to enhance the degree and quality of urbanization of the people, focusing not only on the increase of quantity but also on the improvement of quality. Existing plans should be continuously adjusted and improved to avoid lagging. Positive interaction between planning and process can be achieved, along with the development of targeted strategies in the areas of human population, social welfare, and city facilities according to the coupling, development status, and level of coordination of each city and to create a model of integrated URD with regional characteristics.

## Figures and Tables

**Table 1:** Urban–rural integration and indicator system.

Destination Layer	Criteria Layer	Index Layer	Calculation Method
Level of urban development	Innovation	Technology capital investment	Investment in science and technology as a proportion of fiscal expenditure/%
		Scientific and technical outputs	Number of patents granted
	Coordination	Population urbanization	Number of urban population/Total number of people at year end/%
		Disposable income per urban resident	Income of city residents/Number of urban population/%
		Household Engel coefficient in cities	Coefficient of Urban Engel/%
	Green	Greening coverage of built-up areas	Greening area of built-up area/Area of built-up area/%
		Industrial sulphur dioxide emissions rate	Industrial sulphur dioxide emissions/million tonnes
		Rate of effective discard of household garbage	Harmless treatment rate of domestic waste in the built-up area/%
	Open	Transport network density	Road mileage/administrative land area/km/km^2^
		Internet Density	Number of internet users/urban population/%
	Share	Number of people covered by urban basic health insurance	Number of people covered by urban basic health insurance/person
		Pupil–teacher ratio in city primary schools	Number of urban primary school teachers/primary school pupils/person
		Electricity consumption per million urban residents	Electricity consumption by city residents/number of city residents
Level of rural development	Innovation	Growth rate of food production	Grain growth production/last year’s grain production
		Total farm machinery power per unit area	Total agricultural machinery power/sown area
	Coordination	Level of synchronized industrial development	Increase in the output value of primary production/Increase in the output value of secondary and tertiary production
		Inhabitants of country areas’ disposable income	Disposable income of country inhabitant
		Household Engel coefficient in country	Household Engel coefficient in country
	Green	Effective irrigated area	Effective irrigated area
		Fertilizer application efficiency	Fertilizer application/seeded area
	Open	Transport accessibility	Number of buses per million population
	Share	Number of rural health facilities	Number of health institutions
		Electricity consumption per million rural residents	Rural residential electricity consumption/number of people in villages

**Table 2:** Criteria for classifying levels of coherence.

Level of Coordination	Degree of Coherence (D)	Coordinated Subspecies Types	Level
High degree of coordination	0.90~1.00 (A1)	High-quality coordination	10
	0.80~0.89 (A2)	Good coordination	9
Moderate coordination	0.70~0.79 (B1)	Intermediate coordinate	8
	0.60~0.69 (B2)	Primary coordination	7
Basic coordination	0.50~0.59 (C1)	Barely coordinated	6
	0.40~0.49 (C2)	On the verge of disorder	5
Transitional coordination	0.30~0.39 (D1)	Mild disorder	4
	0.20~0.29 (D2)	Moderate disorder	3
Serious disorders	0.10~0.19 (E1)	Severe disorder	2
	0.00~0.09 (E2)	Extreme imbalance	1

## Data Availability

Data available in a publicly accessible repository.
